# Increased Incidence of Invasive Pneumococcal Disease among Children after COVID-19 Pandemic, England 

**DOI:** 10.3201/eid2808.220304

**Published:** 2022-08

**Authors:** Marta Bertran, Zahin Amin-Chowdhury, Carmen L. Sheppard, Seyi Eletu, Dania V. Zamarreño, Mary E. Ramsay, David Litt, Norman K. Fry, Shamez N. Ladhani

**Affiliations:** UK Health Security Agency, London, UK (M. Bertran, Z. Amin-Chowdhury, C.L. Sheppard, S. Eletu, D.V. Zamarreño, M.E. Ramsay, D. Litt, N.K. Fry, S.N. Ladhani);; St George's University of London, London (S.N. Ladhani)

**Keywords:** invasive pneumococcal disease, bacteria, viruses, respiratory infections, coronavirus disease, pneumococcal diseases, vaccine-preventable diseases, epidemiologic surveillance, pneumococcal vaccines, incidence, COVID-19, SARS-CoV-2, severe acute respiratory syndrome coronavirus 2, England, United Kingdom

## Abstract

During July–December 2021, after COVID-19 restrictions were removed in England, invasive pneumococcal disease incidence in children <15 years of age was higher (1.96/100,000 children) than during the same period in 2020 (0.7/100,000 children) and in prepandemic years 2017–2019 (1.43/100,000 children). Childhood vaccine coverage should be maintained to protect the population.

The COVID-19 pandemic and its associated lockdowns, social isolation, and other interventions led to large declines in respiratory infections, including invasive pneumococcal disease (IPD) (*1*,*2*). In England, IPD cases declined by 30% after the first lockdown in March 2020 and remained low during the subsequent winter until February 2021, when cases increased by 8% above the 3-year prepandemic mean incidence for February (*3*). As the country ended its third national lockdown in March 2021, after emergence of SARS-CoV-2 Alpha variant, IPD cases started to gradually increase. By June 2021, case numbers remained 25% lower than prepandemic levels, but we observed a proportionately higher increase in cases among children <15 years of age (*3*). We describe IPD trends during July–December 2021, after England removed all COVID-19 control measures on July 19, 2021.

## The Study

We compared IPD cases during July–December 2021 to July–December 2020 and July–December in 3 prepandemic years (2017–2019) by using national enhanced surveillance data for England (*2*). In brief, National Health Service (NHS) hospitals electronically report notifiable infections and routinely submit invasive pneumococcal isolates for serotyping to the UK Health Security Agency (UKHSA). For confirmed cases, the UKHSA sends general practitioners a questionnaire regarding risk factors, clinical characteristics, vaccination history, and patient outcomes. To calculate incidence, we used mid-year Office of National Statistics population estimates as denominators, using 2020 data for 2021 because 2021 data were not yet available.

**Figure 1:**
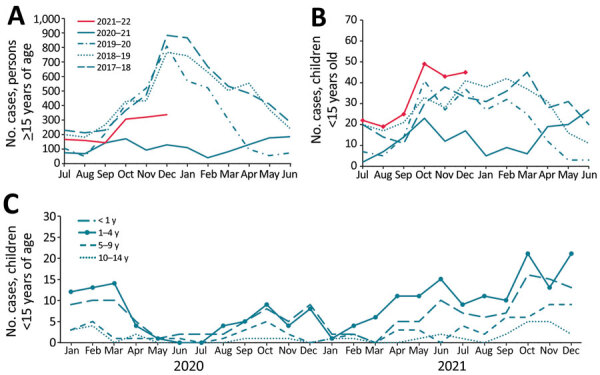
Number of IPD cases among persons before and after COVID-19 pandemic, England. A) Number of IPD cases among persons >15 years of age during July–June by epidemiologic year 2017–18 to 2021–22. B) Number of IPD cases among children <15 years of age during July–June by epidemiologic year 2017–18 to 2021–22. C) Number of IPD cases in children <15 years of age, by month and age group, January 2020–December 2021. IPD, invasive pneumococcal disease.

During July–December 2021, a total of 1,632 IPD cases were reported to UKHSA, compared with a mean of 2,403 during July–December of 3 prepandemic years, 2017–2019 (Figure 1, panels A, B). Among children <15 years of age, the number of IPD and incidence (cases per 100,000 children) declined by 50% (n = 71) during July–December 2020 but gradually increased in February 2021 and remained above the 3-year prepandemic mean of 145 cases (incidence 1.43, 95% CI 1.21–1.68) during July–December 2021 (n = 200; 1.96, 95% CI, 1.70–2.25) (Figure 1, panel B). Case rates rose earlier in younger age groups (Figure 1, panel C) among whom incidence was highest during this period: 10.63 (95% CI 8.19–13.58) among <1-year-olds; 3.22 (95% CI 2.57–3.98) among 1–4-year-olds; 1.02 (95% CI 0.71–1.41) among 5–9-year-olds; and 0.44 (95% CI 0.24–0.72) among 10–14-year-olds. Cases also increased (n = 1,432) among persons >15 years age during February–December 2021 (Figure 1, panel A), but the incidence during July–December 2021 remained lower (2.60, 95% CI 2.47–2.74) than the prepandemic mean during July–December in 2017–2019 (4.14, 95% CI 3.97–4.32).

**Figure 2:**
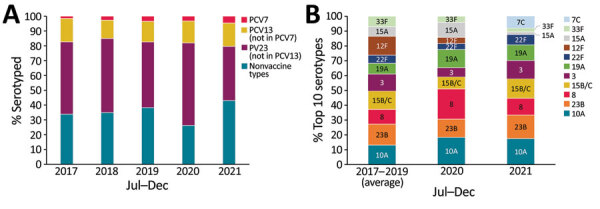
Serotype distribution of pneumococcal isolates from IPD among children <15 years of age, England. A) Percentage of IPD cases by serotype group and year during July–December 2017–2021. PCV7 includes serotypes in the 7-valent PCV; PCV13 includes serotypes in the 13-valent PCV, excluding PCV7 serotypes; PPV23 includes serotypes in the 23-valent pneumococcal polysaccharide vaccine, excluding PCV13 serotypes. B) Top 10 serotypes isolated during July–December 2017–2021. Note these values represent percentages of the top 10 isolated serotypes in each timeframe; the average number of cases of these serotypes compared with all IPD cases was 97/129 for 2017–2019, 49/61 for 2020, and 114/172 for 2021. IPD, invasive pneumococcal disease; PCV, pneumococcal conjugate vaccine.

Age distribution of childhood IPD cases resembled the prepandemic period (p = 0.08): 32% of cases were among <1-year-olds, 42.5% among 1–4-year-olds, 18% among 5–9-year-olds, and 7.5% among 10–14-year-olds. Of 172 (86%) pneumococcal isolates serotyped, we noted no difference in serotype distribution between years nor within age groups. Nonvaccine types (43%) and serotypes in the 23-valent pneumococcal polysaccharide vaccine (PPV23; 37%) but not in the 13-valent pneumococcal conjugate vaccine (PCV13) predominated compared with PCV7 (5%) and additional PCV13 (16%) serotypes (Figure 2, panel A).

The most frequent serotypes among childhood cases remained similar in 2021 to those in prepandemic years (Figure 2, panel A). Of the PCV13 cases, serotypes 3, 19A, and 19F continued to predominate (91% [32/35] compared with 97% [62/64] during the prepandemic period; p = 0.3). Of the additional PPV23 serotypes, the greatest decrease was in serotype 12F, which caused 20% (37/187) of PPV23 cases in the prepandemic period but was not detected during July–December 2021 (Figure 2, panel B). In addition, the proportion of cases attributed to serotype 11A increased from 2% (95% CI 1%–6%; n = 4) prepandemic to 13% (95% CI 7%–24%; n = 8) in 2021. We noted no substantial changes among nonvaccine serotypes.

More IPD cases in 2021 involved bacteremia (50/125; 40%, 95% CI, 32%–49%) compared with the prepandemic period (105/422; 25%, 95% CI 21%–29%) (p = 0.003). The proportion of cases with meningitis (22%), pneumonia (31%), and other clinical manifestations (7%) were not substantially different. The prepandemic and postpandemic 30-day case-fatality rates also were similar (5% vs. 4%; p = 0.6).

## Conclusions

After lifting COVID-19 social restrictions, England experienced an increase in childhood IPD cases that exceeded prepandemic levels. England’s pandemic social restrictions led to large declines in many infectious diseases, including IPD (*1*,*2*). However, a study from Israel reported that pneumococcal carriage in young children declined only slightly during the pandemic (*4*). Reduced social contact and exposure to respiratory pathogens have led to concerns of immunity debt and risk for higher infection rates as restrictions are lifted globally (*5*). Immunity debt is typified in the emergence of respiratory viruses outside their typical season, as observed with respiratory syncytial virus (*6*). Of note, respiratory virus infections that usually peak in winter (e.g., influenza, rhinovirus) remained low during winter 2021–22 (*6*).

Other countries experienced increasing IPD cases after easing national restrictions (*7*,*8*). Germany reported higher IPD rates in children <5 years of age during June–July 2021 than during the prepandemic period (*8*), consistent with our data (Figure 1, panel C). An initial increase among the highest carriage age group that then extends to other age groups was reported with the resurgence of *Haemophilus influenzae* serotype b after mass vaccination in England (*9*), in which the first increases were among 1–3-year-olds. More recently, meningococcal group B disease was highest among university-age students (S. Clark et al., unpub. data, https://doi.org/10.2139/ssrn.3998164), who are the main nasopharyngeal carriers of *Neisseria meningitidis*. Our observed IPD case increase among children is counter to modeling studies that predicted IPD incidence would continue to decline after COVID-19 restrictions were lifted, even accounting for decreased vaccine coverage (*10*). However, these decreases might be because the model did not consider the higher proportion of susceptible children who were not exposed to pneumococci during lockdown (*10*).

Adult IPD cases in England remained lower during 2021 than prepandemic levels. This finding likely is because older adults, who are most at risk for IPD and IPD-related deaths, have continued to socially isolate because of ongoing SARS-CoV-2 infections and emergence of more transmissible variants.

In the United Kingdom, the PCV13 vaccination schedule for infants born after January 1, 2020, was changed from a 2+1 schedule (8 weeks, 16 weeks, and 1 year) that had been in place since 2010 to a reduced 1+1 schedule (12 weeks and 1 year). This change was made on the basis that most protection is through indirect herd or population protection offered by preventing carriage among toddlers, thus interrupting transmission to others (*11*). However, the program relies on maintaining high vaccine coverage in infants to provide adequate population protection. In England, PCV13 coverage data for the 12-month dose were not available for 2020–21 during our study, but uptake of other childhood vaccines was lower after the pandemic started and improved during August–December 2021 (*12*). Because of the COVID-19 pandemic restrictions, evaluation of the effect of the 1+1 schedule is not yet possible.

In our cohort, serotype distribution of childhood IPD cases did not change, consistent with the childhood carriage study in Israel and reports from Germany (*4*,*8*). Switzerland reported an increase in serotype 23B (*7*), but we did not see a major increase in this serotype, although it remains among the most prevalent serotypes responsible for IPD in England.

In conclusion, although total IPD cases remained lower in 2021 than the 3 pre–COVID-19 pandemic years, increases in childhood cases exceeding prepandemic levels could portend increases across all age groups. Maintaining high childhood PCV13 uptake will be critical for ongoing population protection.
